# A critical review of mineral–microbe interaction and co-evolution: mechanisms and applications

**DOI:** 10.1093/nsr/nwac128

**Published:** 2022-07-04

**Authors:** Hailiang Dong, Liuqin Huang, Linduo Zhao, Qiang Zeng, Xiaolei Liu, Yizhi Sheng, Liang Shi, Geng Wu, Hongchen Jiang, Fangru Li, Li Zhang, Dongyi Guo, Gaoyuan Li, Weiguo Hou, Hongyu Chen

**Affiliations:** Center for Geomicrobiology and Biogeochemistry Research, State Key Laboratory of Biogeology and Environmental Geology, China University of Geosciences, Beijing 100083, China; State Key Laboratory of Biogeology and Environmental Geology, China University of Geosciences, Wuhan 430074, China; Illinois Sustainable Technology Center, Illinois State Water Survey, University of Illinois at Urbana-Champaign, Champaign, IL 61820, USA; Center for Geomicrobiology and Biogeochemistry Research, State Key Laboratory of Biogeology and Environmental Geology, China University of Geosciences, Beijing 100083, China; Center for Geomicrobiology and Biogeochemistry Research, State Key Laboratory of Biogeology and Environmental Geology, China University of Geosciences, Beijing 100083, China; Center for Geomicrobiology and Biogeochemistry Research, State Key Laboratory of Biogeology and Environmental Geology, China University of Geosciences, Beijing 100083, China; State Key Laboratory of Biogeology and Environmental Geology, China University of Geosciences, Wuhan 430074, China; State Key Laboratory of Biogeology and Environmental Geology, China University of Geosciences, Wuhan 430074, China; State Key Laboratory of Biogeology and Environmental Geology, China University of Geosciences, Wuhan 430074, China; Center for Geomicrobiology and Biogeochemistry Research, State Key Laboratory of Biogeology and Environmental Geology, China University of Geosciences, Beijing 100083, China; Department of Geology and Environmental Earth Science, Miami University, Oxford, OH 45056, USA; Center for Geomicrobiology and Biogeochemistry Research, State Key Laboratory of Biogeology and Environmental Geology, China University of Geosciences, Beijing 100083, China; Center for Geomicrobiology and Biogeochemistry Research, State Key Laboratory of Biogeology and Environmental Geology, China University of Geosciences, Beijing 100083, China; Center for Geomicrobiology and Biogeochemistry Research, State Key Laboratory of Biogeology and Environmental Geology, China University of Geosciences, Beijing 100083, China; Center for Geomicrobiology and Biogeochemistry Research, State Key Laboratory of Biogeology and Environmental Geology, China University of Geosciences, Beijing 100083, China

**Keywords:** biomineralization, co-evolution, interactions, microbe, mineral, nutrients

## Abstract

Mineral–microbe interactions play important roles in environmental change, biogeochemical cycling of elements and formation of ore deposits. Minerals provide both beneficial (physical and chemical protection, nutrients, and energy) and detrimental (toxic substances and oxidative pressure) effects to microbes, resulting in mineral-specific microbial colonization. Microbes impact dissolution, transformation and precipitation of minerals through their activity, resulting in either genetically controlled or metabolism-induced biomineralization. Through these interactions, minerals and microbes co-evolve through Earth history. Mineral–microbe interactions typically occur at microscopic scale but the effect is often manifested at global scale. Despite advances achieved through decades of research, major questions remain. Four areas are identified for future research: integrating mineral and microbial ecology, establishing mineral biosignatures, linking laboratory mechanistic investigation to field observation, and manipulating mineral–microbe interactions for the benefit of humankind.

## Teaser

Minerals and microbes interact and coevolve through flow of energy and exchange of matter, and their interactions play important roles in driving major geological events and ore deposit formation.

## INTRODUCTION

Minerals are the fundamental Earth materials, and their physical and chemical properties record Earth conditions at the time of their formation. Microbes occupy the majority of the tree of life, and have been dominant players through much of Earth history. In near-surficial environments, minerals and microbes coexist and interact at all spatial and temporal scales. Mineral–microbe interactions are accomplished via flow of energy and exchange of matter. Minerals provide energy [1] and nutrients [2] to support microbial growth and functions. Microbes affect dissolution, transformation and formation of minerals through metabolic activities. These interactions between minerals and microbes substantially determine the habitability of the Earth.

Mineral–microbe interactions leave characteristic signatures in rock records, including morphology, mineral composition and structure, elemental and isotopic fractionation, and organic compounds [3]. These biosignatures demonstrate that minerals and microbes interacted in the past. Since the origination of life on Earth, minerals and microbes have co-evolved over time, and both increased their respective species diversity and functional complexity [2,4]. Mineral–microbe interactions play crucial roles in driving major geological events, such as the Great Oxidation Event (GOE), and formation of ore deposits. With the advent of the Anthropocene Epoch and emergence of human-made minerals, it is important to study consequences of mineral–microbe interactions for the development of a sustainable society.

The topic of mineral–microbe interaction is extremely broad, and spans not only the time interval from the near beginning of the Earth to the present, but also the spatial scale from atoms to globes. There are not only mechanistic investigations on their interactions, but also outcrop-, regional- and global-scale manifestations of such interactions. In light of this breadth, we first describe the pathways and mechanisms of mineral–microbe interactions. Specifically, the role of minerals in supporting microbial activity, and the role of microbial activity in mineral dissolution, transformation and formation are reviewed. We then take geological and environmental perspectives of mineral–microbe interactions over time, highlighting their roles in driving major geological events. Moreover, applications of such interactions are reviewed in the areas of resource recovery, environmental remediation and the carbon cycle. Throughout these topics, we strive to achieve a balance between completeness of coverage and novel insights proposed for future study. At the end, we put forward the grand challenges and future research opportunities in mineral–microbe interaction.

## ROLE OF MINERALS IN AFFECTING MICROBIAL ACTIVITY

**Figure 1.**
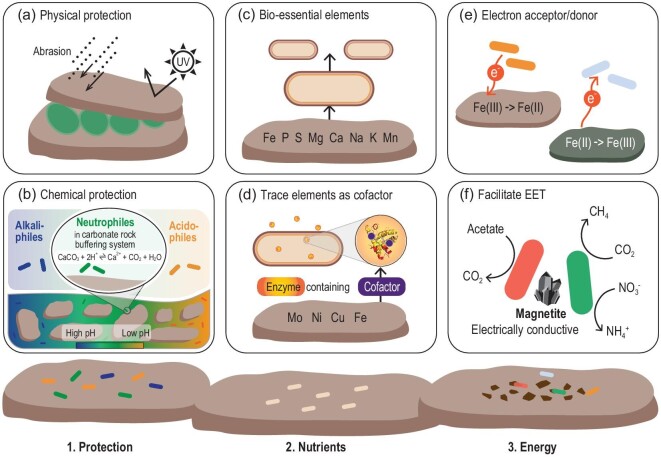
Three roles of minerals in supporting the colonizing microbial community: (1) minerals can offer protection to microbes, including (a) physical and (b) chemical protection; (2) minerals provide nutrients to support microbial growth and metabolism including (c) bio-essential elements and (d) trace metals; (3) minerals provide energy to support microbial growth by serving as (e) electron acceptors/donors and (f) electrical conductors to facilitate extracellular electron transfer (EET).

Microbes nearly colonize all types of mineral surfaces with colonization patterns dependent on the physical and chemical properties of the mineral [5,6]. To emphasize the essential role of minerals, the ecological niche that consists of the minerals and the colonizing microbial communities is defined as the ‘mineralosphere’ [6]. Here, we focus on three beneficial functions of minerals (physical and chemical protection, nutrient supply and energy source, Fig. 1). Minerals not only provide beneficial functions but also exert detrimental effects. We propose two such effects: bio-toxic substances and oxidative pressure of reactive oxygen species (ROS). Previous research has largely focused on the beneficial functions, but has not paid adequate attention to the detrimental effects.

### Protective role of minerals

One fundamental role of minerals is to offer physical protection to microbes. Fractures, fissures and pores within minerals protect microbes from harsh conditions (Fig. 1a), such as UV irradiation, physical abrasion and thermal fluctuation [6,7]. These protective functions of minerals are essential to microbial survival in extreme environments such as deserts, where ecosystems are almost entirely composed of microbial communities in chasmolithic (crevices on rock surfaces), hypolithic (underneath rocks) and endolithic (inside cracks and fissures of rocks) habitats [7,8]. Microbial colonization of the mineral surface is affected by the physicochemical properties of the mineral through electrostatic and hydrophobic interactions. In some cases, mineral surface potential overrides the effect of hydrophobicity in controlling microbial colonization. Negatively charged microbial cells attach not only to a positively charged mineral surface but also to a negatively charged one, because they need nutrients from minerals and their cell walls possess specific functional groups to form chemical bonds with the mineral surface [6]. Once attached, the amount of structural Fe in minerals is an important parameter in determining the extent of the UV shielding capacity [9]. Many minerals have the ability to offer protection against various environmental stresses, but the physicochemical factors that determine the level of protection have not been systematically evaluated, which is important for understanding microbial survival strategies in extreme environments, early Earth and on extraterrestrial planets.

Minerals also offer chemical protection by creating specific micro-environments to favor certain microbial populations. This role is exemplified by the colonization of microbes on alkaline or acidic habitats of a mineral surface (Fig. 1b). In serpentinizing peridotite, mineral surface pH becomes alkaline, and alkaliphilic microorganisms thrive in these habitats. Conversely, oxidation of metal sulfides creates acidic pH, where acidophilic microbes are found. Certain minerals with a pH buffering capacity, such as carbonates, can harbor neutrophilic sulfur-oxidizing microorganisms, because acidity created by sulfur oxidation can be neutralized by carbonate minerals [10].

### Minerals as sources of bio-essential elements and enzyme metal cofactors

Minerals and rocks not only provide protection to microbes but also supply nutrients to support their metabolism (Fig. 1c and d). Most bio-essential elements required to sustain microbial growth and metabolism are originally sourced from minerals [11]. Therefore, mineral colonization may be a chemotactic process (response to a chemical gradient) driven by nutrient availability. Minerals and rocks are especially important to microbial growth under oligotrophic conditions, such as in granite or basalt habitats. A common strategy for microorganisms to extract bio-essential elements from minerals and rocks is through production of metabolites to enhance mineral dissolution [12]. During dissolution, a passivating layer may form at the surface of a mineral to slow down the rate of dissolution [13], which may be beneficial to the microbes because bio-essential elements may be less depleted by a chemical process (such as fluid transport).

The importance of minerals as a nutrient source is evidenced by their impact on microbial community structure and functions. Laboratory and field studies (summarized in Table S1) show that microbial communities vary in relation to the elemental composition of minerals, especially Fe and P [14,15]. Other elements, including Na, Si, Mn, S, Mg, Ca and K, are also important [6] (Table S1). However, the presence of certain elements in minerals does not necessarily suggest bioavailability. Thus, the mineral weatherability index is used to estimate the bioavailability of nutrients in minerals (Table S1). A significant correlation was found between apatite weatherability and the abundance of certain bacteria (*Betaproteobacteria*) colonizing the apatite surface [16]. Stable minerals and rocks (i.e. resistant to weathering, such as apatite and obsidian) tend to attract effective mineral-weathering bacteria relative to easily weathered minerals (i.e. calcite) [17]. With advancements in transcriptomics and proteomics, both laboratory and field studies have identified differential gene expressions and physiological responses to mineral additions (Table S1). These responses are likely to enhance mineral weathering and nutrient acquisition from mineral structure.

In addition to bio-essential/major elements, minerals also provide trace metals as integral components of biological catalysts (i.e. metal-containing enzymes called metalloenzymes) (Table S1). These enzymes require active metal centers (called metal cofactors) to function properly. **Mo** is an essential metal cofactor for the activity of Mo-based nitrogenase, an enzyme that reduces N_2_ gas to bioavailable NH_3_ by N_2_-fixing bacteria. Field studies found that Mo limitation can slow down the nitrogen fixation process [18], and as a result, N_2_-fixing bacteria may switch to alternative, less-efficient nitrogenases using Fe and V as metal cofactors [19]. Some N_2_-fixing bacteria may be able to extract Mo from silicate glass [20] to compensate for the deficiency of aqueous Mo in natural environments.

Another example of a metal cofactor is Ni, which, along with Fe and Co, is required for formation of greenhouse gas methane (i.e. methanogenesis). Low bioavailability of Ni limits methanogenesis, and the addition of Fe, Ni and Co to metal-deficient peatlands results in enhancement of methane production [21]. The ‘Cu switch’ of methane-oxidizing microbes (called methanotrophs) is another example of metal cofactor availability in minerals [22]. The availability of Cu can regulate production of methanobactin (a Cu-binding ligand) and the level of a methane-oxidizing enzyme called methane monooxygenase (MMO). Therefore, MMO level and methane oxidation capacity are largely controlled by Cu mineralogy when aqueous Cu is scarce [23]. The bioavailability of Cu in mineral structures impacts methane oxidation rate and methanobactin production by the methanotrophic bacterium *Methylosinus trichosporium* OB3b [24]. Interestingly, methanobactin (and other metabolites) may be used by a microbial community as ‘public goods’ for copper acquisition [25].

### Minerals as a source of energy

Another important function of minerals is to provide energy to microbes through redox reactions of elements, especially Fe, S and Mn. Redox-active minerals can support microbial growth as: (i) an electron sink or source; (ii) an electrical conductor to facilitate extracellular electron transfer; (iii) an environmental battery; and (iv) a photocatalyst to stimulate photosynthesis [1].

Given that Fe-containing minerals are ubiquitous in the environment, microbial populations have a capacity of exploiting the energy released from Fe redox reactions [26,27]. These microorganisms include Fe(II)-oxidizing autotrophs and Fe(III)-reducing heterotrophs [26]. Microbes are also capable of deriving energy from sulfur redox reactions. Certain bacteria can derive energy from oxidation of sulfide minerals [28] and reduction of sulfate minerals [29]. Unlike Fe and S, the majority of biological Mn oxidation does not generate much energy, despite the wide distribution of Mn-bearing minerals and Mn(II)-oxidizing microorganisms in nature [30,31]. Likewise, only a few researchers reported cell growth with Mn(IV) as the sole electron acceptor [30,31]. However, diverse microbes, dominated by Mn-reducing and Mn-oxidizing bacteria, are present in Mn nodules and ferromanganese crusts, implying the existence of microbial communities possibly fueled by Mn minerals.

Certain metal sulfides and oxides have long been recognized as electrical conductors that facilitate electron transfer. Many iron-containing minerals mediate electron transfer between microbes of the same or different species [1]. For example, hematite, magnetite and iron sulfides all exhibit a capacity to promote methanogenesis by acting as a conduit between methanogen and Fe(III)-reducing species [32]. Similar results are reported in studies in which syntrophy occurs between Fe-reducing bacteria and nitrate- or sulfate-reducing bacteria due to the presence of these minerals [32].

Redox-active minerals function as environmental batteries. Magnetite is reversibly oxidized and reduced by a co-culture of Fe(II)-oxidizing and Fe(III)-reducing bacteria [33]. Thus, two types of microbes, one using Fe(III) as an electron sink and the other using Fe(II) as an electron source, can both gain energy even when they are spatially and/or temporally separated. Other mixed-valence Fe minerals, including green rust and iron-containing clays, have the potential to support microbial syntrophic metabolism by functioning as environmental batteries [1,34].

Finally, phototrophic bacteria use electrons of electrically conductive metal oxides to fix CO_2_ [35]. For some microbes that are unable to use sunlight directly, light-sensitive and semi-conductive minerals can use sunlight as an energy source to transfer electrons to these microbes for energy acquisition. For example, some non-phototrophic microbes can obtain energy from the photoelectrons generated by solar irradiation of semi-conductive metal sulfides and oxides [36]. These examples highlight a wide range of strategies that microbes employ to harness energy from minerals.

### Minerals as sources of bio-toxic substances

Minerals can affect the colonizing microbial communities through accidental release of bio-toxic substances from minerals. The majority of minerals contain metals, including ones that, at low levels, are essential for life but at high levels are toxic (e.g. Cu, Zn, Co and Mn), and others that are toxic at all levels (e.g. Cs, Al, Hg and Pb). When microbes acquire nutrients, they are inevitably exposed to toxic metals. To cope with metal toxicity, microbes develop various strategies to decrease metal bioavailability by changing their speciation [37]. Specific strategies include biosorption of metals to biofilm, cell wall and extracellular polymeric substance (EPS); precipitation as minerals; redox reactions [e.g. Hg(II)→Hg(0)] [11]; and metal sequestration by binding to proteins/ligands [38]. Other mechanisms include expulsion of metals by permeability barrier, active efflux and suppression of influx [39].

### Minerals as sources of oxidative pressure

A number of metal-bearing minerals can produce ROS through dissolution and release of metal ions, redox reactions and surface defects [40]. The generation of ROS depends on the physicochemical properties of minerals and environmental conditions (such as UV wavelength, solution pH and O_2_). Although a base level of ROS may be required to support life, higher levels can degrade essential biological molecules including DNA, RNA and proteins, because of their strong oxidizing nature [41]. As a defense mechanism, microbes produce antioxidant enzymes to clear up ROS such as superoxide dismutase, catalase/peroxidase, peroxiredoxin and ferric reductase/oxidoreductase [41]. However, the activity of these enzymes requires metal cofactors, which are ultimately derived from minerals. Thus, minerals serve as a double-edged sword, having both detrimental and beneficial effects on microbial activity.

Nonetheless, ROS may ultimately kill certain populations of a microbial community while genetically mutating others. Current research is limited to the antibacterial activity of ROS [42], but how mineral-derived ROS change microbial community structure and function is poorly known. More importantly, ROS-induced genetic mutation may increase functional diversity. Therefore, mineral-induced ROS may represent an important selection pressure to drive microbial evolution, which represents an important area of future study.

For clarity, the beneficial and detrimental effects of minerals are individually described above, but in reality, these effects occur simultaneously. In a microbial population, there is division of work among different members. Some members may acquire either nutrients or energy from minerals to share with others as ‘public goods’, while others may develop defense mechanisms to mitigate environmental stress. To effectively coordinate these complex interactions, cell-to-cell signaling, regulated gene expression and horizontal gene transfer may be crucial to maintain a sustainable community. These interactions may be more active within a mineral-attached biofilm than within a planktonic community, because of more efficient communication and more stable community structure (i.e. less sensitive to environmental perturbation) within a biofilm. Future investigations are warranted to study syntrophic and antagonistic interactions among different members of a community, especially within a mineral-attached biofilm community.

## ROLE OF MICROBIAL ACTIVITY IN MINERALIZATION

**Figure 2.**
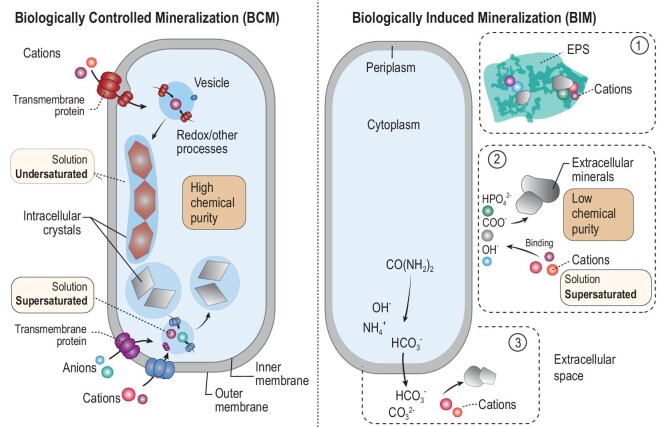
Schematic diagrams of biologically controlled and induced mineralization (BCM and BIM). In BCM, anions and cations are taken up through transmembrane proteins into the cell. Through genetically controlled biochemical processes, minerals reach supersaturation and precipitate inside a membrane, despite extracellular undersaturation. In BIM, minerals of relatively non-uniform size and low chemical purity form via three possible pathways: (1) anionic species of extracellular polymeric substances (EPSs) bind with cations to nucleate mineral formation; (2) anions on the cell surface serve a similar function to nucleate mineral formation; (3) anionic species are excreted to outside the cell to bind with cations to precipitate minerals.

Microbes are not passive recipients of energy and nutrients from minerals. Instead, they actively dissolve, precipitate and transform minerals through metabolism [43]. Because microbes dissolve/weather minerals to acquire nutrients, the microbial role in mineral weathering and dissolution is already described in the previous section. Here we emphasize the role of microbes in mineral precipitation and transformation, a process termed biomineralization. The biomineralization pathway is broadly classified into two types: biologically controlled and induced mineralization (BCM and BIM) (Fig. 2).

BCM is strictly controlled by a set of genes that regulate import of elements/ions, intracellular biochemical processes and the formation of crystals inside the cell [44]. The extracellular environment is usually undersaturated, but the intracellular environment is oversaturated to precipitate the mineral. The minerals formed through BCM often have a uniform size distribution, high chemical purity (Fig. 2) and specific functions. In contrast, BIM occurs when biological activity alters the surrounding environment such that certain minerals become oversaturated and precipitate outside the cell [45]. The BIM process initiates when cations are electrostatically attracted to negatively charged functional groups of the cell surface or within EPSs (Fig. 2). The minerals formed through BIM may have characteristic morphologies and organic compounds. The resulting minerals were once thought to have no utility, but recent evidence points to their physiological functions. Through biomineralization, biogenic minerals may contain diagnostic features that can be used as signatures to infer past mineral–microbe interactions.

A related biomineralization pathway, termed biologically influenced mineralization, refers to mineral precipitation onto biological structures from an oversaturated solution. In this pathway, mineral precipitation itself is an abiotic process, but biological structures (such as EPSs and cell walls) serve as templates or nucleation surfaces to influence mineral morphology and chemical composition. Unlike the induced pathway, the influenced pathway does not require the presence of active cells or enzymes. In this review, we do not specifically distinguish between induced and influenced pathways.

### Biologically controlled mineralization

The major types of minerals formed via BCM are magnetite, greigite, mackinawite, Ca-carbonates, elemental S^0^ and certain phosphate minerals (e.g. struvite and hazenite). This short list does not necessarily suggest shortage of BCM minerals, but may reflect lack of efforts and/or techniques to identify them. The BCM process can be catalyzed by prokaryotic and eukaryotic microbes across diverse environmental habitats.

Intracellular chains of magnetite and greigite crystals (called magnetosomes) are currently the best-known examples of BCM made by magnetotactic bacteria (movement in response to Earth's magnetic field). The synthesis of these minerals involves a complicated sequence of genetically controlled steps, including cellular uptake of Fe, formation of magnetosome membrane and growth of crystals into a chain [44,46]. These crystals have a narrow size range (35–120 nm) and characteristic shape, and are generally free of chemical impurities. One function of magnetic crystals is to help the host cell find an optimal location along the O_2_ gradient in an aquatic environment [44]. Magnetosomes may have certain enzymatic activity to degrade intracellular ROS. This activity may have been critical to the survival and evolution of magnetotactic bacteria on early Earth, when the atmospheric O_2_ level gradually increased [46].

Another example of BCM is intracellular carbonates. Amorphous calcium carbonates (ACCs) form through active uptake of Ca and formation of a membrane [47,48], which may be controlled by specific genes [49]. ACCs share some characteristics of the minerals found in magnetosomes, such as a narrow size range and a membrane surrounding these crystals. Unlike magnetosomes, however, ACCs are amorphous and contain certain chemical impurities (Mg, Sr and Ba) [50], suggesting preferential co-uptake of these elements from aqueous solution. Because their formation needs energy input, ACCs are speculated to possess specific functions as: (i) an inorganic carbon storage reservoir for CO_2_-fixing microorganisms; and (ii) a buffer of pH change caused by oxygenic photosynthesis and/or sulfur oxidation [47]. The presence of ACC modifies the buoyancy via gravitaxis (movement of an organism in response to gravity). The formation of both ACCs and magnetosomes in the same magnetotactic bacterium [51] may equip the host with more versatile functions, such as control of motility via magnetotaxis and direction via gravitaxis. As more effort is underway, we are likely to find more BCM minerals and new functions.

### Biologically induced mineralization

Compared to the limited number of BCM minerals, the number of BIM minerals is much more diverse, including all major groups of minerals [43,45,52,53]. Some of these mineral formations are induced by specific microbes, while others are made by diverse types of microbes. Here we describe a few examples of geologically important mineral transformations/reactions.

Microbially catalyzed Fe redox processes have received much attention because they produce not only energy to support microbial activity but also diverse BIM minerals. For details, readers are referred to recent reviews [1,26,27,43,52,54]. Here we focus on mineral transformations induced by microbial Fe(III) reduction and Fe(II) oxidation.

Microbially catalyzed Fe(III) reduction induces many mineral reactions. One important example is the smectite-to-illite transformation, which is considered to be one of the most important mineral reactions during sediment diagenesis, as the degree of this reaction, termed ‘smectite illitization’, is linked to maturation, migration and trapping of hydrocarbons in sedimentary basins. This reaction had been believed to be only a function of geological conditions (temperature, pressure, time, K availability, pH and water/rock ratio) until the discovery of the microbial role. Through reduction of structural Fe(III) in smectite, microorganisms can promote the smectite-to-illite reaction at room temperature and one atmospheric pressure within two weeks [55], which is in sharp contrast to the abiotic reaction. More laboratory and field evidence has accumulated to support the microbial role in this reaction [56,57]. Laboratory investigations have focused on diverse microorganisms under geologically relevant conditions such as thermophilic bacteria, high pressure and organic matter. Field studies have increasingly recognized the microbial role in catalyzing illite formation in K-bentonites on the Permian-Triassic boundary [58], in the Proterozoic carbonates [59]; Nankai Trough mudstones [57] and sedimentary rocks of ∼2.1 Ga in age [56].

The other half of the Fe redox cycle is Fe(II) oxidation. Upon oxidation of aqueous Fe(II), Fe(II) oxidizers can produce a variety of (oxy)hydroxides including ferrihydrite, goethite, lepidocrocite, akageneite and green rust [26,27]. These minerals vary in morphology, composition and structure. By oxidation of structural Fe(II) in illite, Fe(II) oxidizers can drive the illite-to-smectite reaction [60]. This example illustrates the fact that when the driving force reverses [i.e. Fe(II) oxidation as opposed to Fe(III) reduction], the resulting mineral reaction also reverses (i.e. the illite-to-smectite reaction as opposed to the smectite-to-illite transformation). Thus, different microbes are capable of catalyzing the forward and reverse reactions, and their roles should be taken into account when geothermometer models are developed based on clay mineral reactions [61].

Another well-studied BIM example is dolomite [CaMg(CO_3_)_2_]. The physical and chemical properties of dolomite rocks in sedimentary basins determine the size and quality of oil reservoirs. Sequestration of CO_2_ into dolomite rocks may have partially contributed to the gradual cooling of the Earth to the extent that life became possible. Thus, understanding the mechanisms of dolomite formation may help achieve a carbon-neutral economy. However, the mechanism of dolomite formation has remained enigmatic. Efforts to synthesize dolomite in the laboratory under simulated conditions (i.e. low temperature and pressure) have largely failed. Dolomite formation is thermodynamically favorable, but kinetically slow. The hypothesis is that Mg^2+^ forms ion pairs with sulfate or is strongly hydrated by water molecules, which makes Mg^2+^ unavailable for dolomite precipitation.

A discovery was made when Vasconcelos and colleagues successfully precipitated dolomite in culture experiments in the presence of sulfate-reducing bacteria [62]. These bacteria can overcome the kinetic energy barrier by increasing pH and carbonate alkalinity and by removing sulfate, a known inhibitor to dolomite formation. Later studies demonstrated that sulfate reducing bacteria (SRB), halophiles, sulfide oxidizers, methanogens and even microbial EPSs/cell walls can all catalyze dolomite formation [63]. Abiotic precipitation of dolomite is even possible, as long as Mg^2+^ is released from ion pairs or a hydrated state [64,65]. Therefore, the microbial role in dolomite formation appears to be primarily freeing Mg^2+^ from Mg^2+^-H_2_O and MgSO_4_ complexes via removal of water and/or sulfate. Biogenic dolomite is disordered in crystal structure, but ordered dolomite is often observed in geological records. Clearly, more evidence is necessary to distinguish the enzymatic and non-enzymatic role of microbes in precipitating dolomite under natural low-temperature conditions, including multiple microbial species and complex aqueous chemistry.

Although BCM and BIM are well-established, there may be hybrid pathways that fall between the two, including grains of iron oxides inside *Shewanella putrefaciens* [66] and Fe-, As-, Mn-, Au-, Se- and Cd-precipitates within diverse bacteria [67]. Enzyme-mediated mineral precipitation may be another example of a hybrid BCM–BIM pathway. For example, phosphoryl groups liberated from acid phosphatases can combine with uranium to precipitate uranium phosphate [68].

Regardless of either BCM or BIM, much of the current research is focused on prokaryotes (mostly bacteria), but the role of eukaryotes in biomineralization is under-studied. Possible reasons are the early evolution of bacteria, their active role in catalyzing redox reactions, and thus their perceived importance in biomineralization. However, the role of certain eukaryotes (especially fungi) has been increasingly recognized [69], especially in the soil rhizosphere, where fungi and plants form a syntrophic relationship. Many minerals can be produced by fungi, including metal oxalates, carbonates, iron and manganese oxides, metal formates and even the high-temperature mineral olivine (forsterite, Mg_2_SO_4_) [69,70].

### Mineral biosignatures in the geological record

Unlike macrofossils, microbes usually cannot be fossilized to recognizable morphology. However, biomineralization may enhance the fossilization potential of cellular structures and preservation of organic molecules [71]. Nonetheless, inference of past mineral–microbe interactions heavily relies on mineral biosignatures [3,72,73]. However, establishment of such biosignatures is not straightforward, because biogenic and abiogenic minerals share some similar characteristics.

A proposed example of a biogenic mineral is intracellular magnetite crystals. Thomas-Keprta and colleagues [74] proposed six criteria for the biogenicity of these crystals, including single-domain size and restricted width/length ratios, chemical purity, crystallographic perfection, magnetite chains, unusual crystal morphology and crystallographic direction of elongation of magnetite crystals. The authors used these criteria to argue for the biogenicity of some magnetite crystals in a Martian meteorite. However, subsequent studies [75] suggested that shock metamorphism could form similar magnetite crystals. Thus, additional characteristics would strengthen the biogenicity claim, including lower levels of trace element incorporation into magnetite crystals and specific magnetic properties [76]. Compared to BCM minerals, biogenic features in BIM minerals are even less diagnostic. However, some BIM minerals have distinct morphologies such as twisted stalks [77], and tubular and granular textures [78,79] that have few abiotic equivalents. Moreover, organic compounds preserved within minerals [80] may be used as additional support for their biological origin.

Individual minerals may not be so diagnostic of biogenicity, but assemblages of multiple minerals may be more convincing [81]. Additional evidence may include morphology [82], crystal structure, trace and rare earth element composition [83,84], isotopic fractionation (especially metal isotopes), and structural incorporation of organic molecules. For some minerals, clumped isotopes may be useful to help constrain the temperature of mineral formation [85]. Spatially resolved isotopes (i.e. isotopic composition of intracellular versus extracellular crystals) are a powerful tool to supplement mineralogical evidence. Even with these approaches, a critical question remains with regard to the preservation potential of mineral biosignatures. Biogenic minerals such as ferrihydrite and goethite are often poorly crystalline and metastable, and transform to more stable hematite and magnetite under elevated temperatures, but the mineralized textures (such as stalks and cellular shapes) may be better preserved [79,86]. Therefore, multiple lines of evidence should be sought to establish the biogenicity of minerals in geological records, which is fundamental to the investigation of mineral–microbe co-evolution.

## MINERAL–MICROBE CO-EVOLUTION

**Figure 3.**
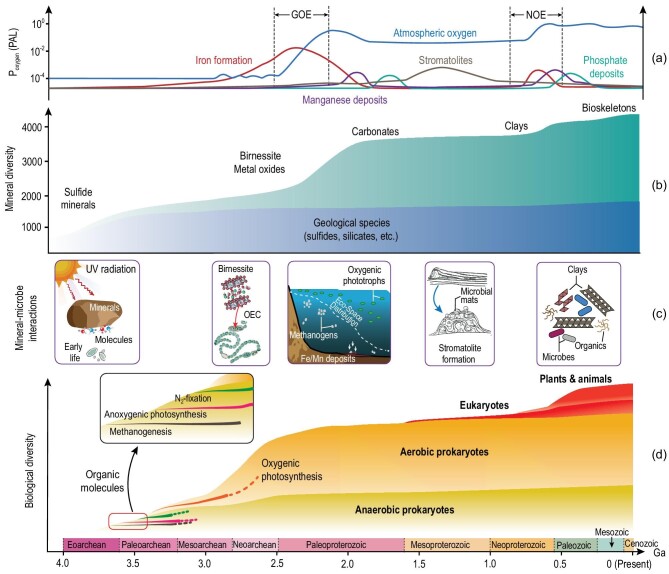
Mineral–microbe co-evolution. (a) Evolution curves of atmospheric oxygen content [89], iron formations [90], Mn deposit [91] and phosphorite deposit [92]. Mineral–microbe interactions play important roles in sequential formation of Fe, Mn and P deposits around the Great Oxidation Event (GOE) and the Neoproterozoic Oxygenation Event (NOE). PAL: present atmospheric level. (b) Simplified mineral evolution sequence showing important groups of minerals [4]. The geological species refer to those high temperature minerals that were present before the origination of life. The green shade refers to those that have interactions with life. The positions of these major groups of minerals in the green shade denote the approximate times their diversity increases. The sum of the two categories is approximately equal to the total number of mineral species. (c) A few important examples of mineral–microbe interactions over time. On early Earth, sulfide minerals serve as catalysts and protectants of molecules and early life. Mineral–microbe interactions drive the GOE through (i) the role of a birnessite-like mineral in origination of oxygenic photosynthesis through its catalytic function in the oxygen evolving complex (OEC); (ii) a decrease of toxic ROS production through anaerobic oxidation of Fe(II) to Fe(III) minerals so that oxygenic phototrophs flourish; (iii) time-course expansion of the eco-space of oxygenic phototrophs and shrinkage of methanogens. Mineral–microbe interactions are also important in stromatolite formation during the Boring Billion (mid-Proterozoic). Around the NOE, sequestration of organic carbon by clay minerals results in a further rise of O_2_. (d) Evolution of a few important groups of microorganisms. Biological diversity is only schematic. The time of eukaryote emergence is based on Ref. [93]. Eukaryotes (the whole red-shaded region) include plants and animals (the upper portion of the red-shaded region). The boundaries among different geological time units are based on the latest published data (https://stratigraphy.org/ICSchart/ChronostratChart2022-02.pdf).

Mineral–microbe interactions have not only occurred in modern environments but also over Earth history, i.e. co-evolution (Fig. 3). At the Earth’s beginning, there were only a few dozen high-temperature minerals (geological species), and even with subsequent igneous activity, metamorphism and plate tectonics, the number of mineral species only increased to ∼1500 [4]. After the GOE, the mineral species increased to >4000. The origination of eukaryotic organisms led to the formation of some organo-minerals, with the total number of mineral species reaching a modern level of >5000 [4]. Likewise, microorganisms experienced a long history of evolution, from obligate anaerobes on early Earth to many aerobes in modern environments [2]. Throughout Earth history, mineral–microbe interactions have widely occurred at the microscopic scale but the effect is often seen at the global scale. Here we take geological and environmental perspectives to examine mineral–microbe co-evolution (Fig. 3), highlighting their roles in driving major geological events including the GOE and ore deposit formation. However, it is important to recognize that this subject is poorly known, because of insufficient identification of biosignatures in geological records and limited laboratory simulation studies.

### Role of minerals in catalysis/protection of organic molecules and origination of life

Due to strong UV irradiation and other harsh conditions on early Earth, the protective role of minerals was essential for preserving and concentrating prebiotic organic molecules to initiate primitive biochemical reactions. Minerals, especially clay minerals, serve as catalysts and scaffolds to polymerize organic monomers into macromolecules such as proteins and nucleic acids [87,88]. Some minerals, such as calcite, may even play a role in selecting one of the enantiomeric amino acids and nucleotides [87]. Montmorillonite can interact with fatty acids to form protocells with the abilities of growth and division [94]. After life origination around 3.7 Ga [95], minerals continued to serve as protectants of life itself [8,9,96].

### Role of mineral evolution in driving microbial innovation

Since life origination, minerals and microbes have co-evolved through much of Earth history. The mineral weatherability index and bioavailability of nutrients generally increased over time, which would drive microbial evolution. However, this subject is poorly understood in general and we use two examples to only illustrate the concept.

The first example is co-evolution of iron sulfide minerals and biological Fe-S clusters. Certain iron sulfide minerals can catalyze various biochemical reactions [97]. Interestingly, ancient organisms, such as methanogens and thermophiles, contain intracellular Fe-S clusters, which play important roles in biochemical functions such as electron transfer and DNA repair [98–100]. Therefore, ancient organisms may have the ability to assimilate iron sulfide minerals (such as greigite) into cells to perform catalytic functions. This scenario is supported by the high structural similarity of iron sulfide minerals and biological Fe-S clusters [101]. Over time, iron sulfides are oxidized to form insoluble iron oxides and soluble sulfate, and consequently, synthesis of biological Fe-S clusters involves the more complicated steps of acquisition of Fe and S, intracellular redox reactions, and cluster biogenesis [102].

The second example is the role of mineral-associated metal cofactors in microbial evolution. In Archaean oceans, enzymes likely used soluble Fe as a cofactor, because of its high abundance [103]. As minerals evolved as a result of rising O_2_, other metals, including Cu, Zn, Mo and Ni, became more bioavailable [103]. Thus, the different types of microbial metabolisms, which depend on metal cofactor bioavailability, greatly diversified [2]. For the same metabolic function, bioavailability of more metals would increase the efficiency of that function. For example, when Mo became more bioavailable after Mo-bearing minerals evolved from sulfides to oxides and molybdates, Mo would replace Fe as a more efficient cofactor for N_2_ fixation. However, isotopic [104] and phylogenetic evidence [105] suggests that N_2_-fixing bacteria were able to use Mo as cofactors even in Archaean oceans when most Mo was locked up in sulfide minerals such as molybdenite. Therefore, these ancient anaerobes may have mechanisms to acquire Mo from minerals of low solubility and bioavailability (Fig. 3). However, this speculation requires rigorous testing.

### Role of microbial evolution in driving mineral diversification

Molecular O_2_, which is a product of oxygenic photosynthesis, is a major driver of mineral evolution [4]. Thus, most of the minerals today are an indirect consequence of microbial activity. Here we limit the discussion to the direct consequence of microbial evolution on mineral diversification. Again, this area is poorly known and we illustrate the idea through two examples: (i) Fe(II) oxidation and (ii) methane oxidation. Because these metabolisms exhibit an anaerobic-to-aerobic transition over time, assemblages of biominerals and their functions show co-evolution as a result.

The first example is Fe(II) oxidation, which consists of four pathways roughly relating to the order of their origination: (i) the ancient nitrate-dependent anoxic Fe(II) oxidation; (ii) the anoxygenic phototrophic Fe(II) oxidation before the GOE; (iii) the neutrophilic and microaerophilic Fe(II) oxidation before the GOE; (iv) acidophilic Fe(II) oxidation after the GOE [106]. Nitrate-dependent Fe(II) oxidation produces mineral assemblages of ferrihydrite, goethite and green rust [26,27]. Anoxygenic phototrophic Fe(II) oxidizers produce ferrihydrite on cell surfaces to offer protection against harmful UV radiation on early Earth [96]. Microaerophilic Fe(II) oxidizers produce ferrihydrite, lepidocrocite and goethite along twisted stalks and tubular sheaths to help them find optimal niches along opposing O_2_ and Fe(II) gradients [107] and protect them from toxic ROS [108]. Localization of biogenic iron oxyhydroxides in the vicinity of the cells may help establish a proton motive force for energy generation [77]. This example illustrates that as Fe(II) oxidation pathways evolved, both the identity of iron (oxy)hydroxide minerals and their functions co-evolved.

The second example is methane oxidation. The evolutionary history of methanotrophs is not well-established, but anaerobic organisms with Fe(III), Mn(IV) and sulfate as electron acceptors should predate aerobic ones, possibly with nitrite-driven, oxygenic methane-oxidizing bacteria (‘*Candidatus* Methylomirabilis oxyfera’) as an intermediate [109]. Therefore, as methanotrophs evolved from anaerobic to aerobic, dominant mineral products likely evolved from assemblages of iron/manganese sulfides, elemental sulfur, sulfate minerals and iron- and manganese-bearing carbonates to calcium- and magnesium-bearing carbonates. Additional examples may be found in other microorganisms that show anaerobic-to-aerobic transition such as sulfur-oxidizing and ammonia-oxidizing bacteria [including aerobic ammonia oxidation, anaerobic ammonia oxidation with nitrite, Fe(III) and Mn(IV) as electron acceptors].

### Role of mineral–microbe interactions in driving major geological events

#### The Great Oxidation Event

Oxygenic photosynthesis is believed to have emerged more than 3.0 Ga ago [110,111]. However, there is evidence for a more recent origin, not earlier than 2.5–2.6 Ga [112]. Nevertheless, it is likely that there is a time gap between cyanobacteria emergence and oxygenation of the Earth’s atmosphere [89]. The exact timing of Earth oxygenation (i.e. the GOE) is determined by a balance between production and consumption of O_2_ [89]. In this oxygenation process, mineral–microbe interactions played an important role, although some of these roles may be indirect.

First, a manganese oxide mineral, birnessite, may have played a key role in the origination of oxygenic photosynthesis. All oxygenic phototrophs contain the Mn_4_CaO_5_ cluster in their oxygen evolving complex (OEC). However, it is only after the transformation of this cluster to a birnessite-like structure that these organisms are able to produce free O_2_ [113]. Interestingly, birnessite itself is known to capture sunlight for water-splitting catalysis [114] and such Mn oxides were already present before the rise of oxygenic photosynthesis [115]. Therefore, the photocatalytic oxygen production by layered Mn oxides may be the embryonic form of biological photosynthesis [116]. However, the genetic connection between Mn oxide minerals and the origin of oxygenic photosynthesis remains an area of future research.

Second, once free O_2_ is present in the shallow oceans, soluble Fe(II) reacts with O_2_ to form highly reactive ROS, which are toxic to phototrophs and would limit their proliferation [117]. However, some bacteria can synthesize nanometer-sized magnetite to lower the intracellular ROS level [46]. Furthermore, anaerobic oxidation of aqueous Fe(II) to iron oxides by Fe(II)-oxidizing bacteria not only lowers Fe(II) concentration in seawater but also decreases O_2_ consumption and ROS production, all of which favor O_2_ accumulation.

Third, the presence of free O_2_ increases mineral weathering [4,118,119] and metal cofactor bioavailability (such as Mo), which expands the eco-space of oxygenic phototrophs through prevision of fixed nitrogen and other nutrients (i.e. P). At the same time, a diminished supply of Ni from the mantle source weakens methanogenesis [120,121]. Consequently, the expansion of eco-space for oxygenic phototrophs and diminished eco-space for methanogens favors O_2_ accumulation. Moreover, the evolution of more bioavailable Cu minerals would enhance aerobic methanotrophy, which reduces methane flux and increases the O_2_ level in the atmosphere.

The combination of these processes drives the ultimate occurrence of the GOE. In return, the GOE leads to diversification of mineral and microbial species. After the GOE, many oxidized metal (oxy)hydroxide minerals formed [4], which are generally more bioavailable to promote synthesis of new metalloenzymes for aerobic metabolisms [2].

#### Formation of iron, manganese and phosphorous ore deposits

Another direct consequence of mineral–microbe co-evolution is the sequential formation of iron, manganese and phosphorous deposits around two episodes [approximately corresponding to the GOE and the Neoproterozoic Oxygenation Event (NOE)] (Fig. 3). The formation of these deposits is controlled by a combination of source input, microbial activity, O_2_ level and seawater chemistry.

Banded iron formation (BIF) is the most important source of Fe ore. BIF consists of alternating layers of iron oxides (magnetite and hematite) and chert. The first BIF occurred as early as 3.8 Ga, reached peak abundance between 2.7 and 2.4 Ga (before the GOE), and lasted until 1.8 Ga. There are minor occurrences of granular iron formation (GIF) in the Mesoproterozoic and Neoproterozoic [121]. Recent evidence suggests the important role of microbial Fe redox cycling in the origin of BIF [121].

Mn deposits mostly occur after BIF and GIF in the forms of carbonates and mixed-valence silicates (rhodochrosite and braunite) [91], apparently as a result of the Mn redox cycle [122]. Oxidation of Mn(II) by molecular O_2_ is slow at neutral pH, and most Mn(II) oxidation is catalyzed by aerobic bacteria and fungi [30,123] via multi-copper oxidases. Mn(II) oxidation catalyzed by iron oxide surface is also possible [124]. Because biological Mn(II) oxidation depends on availability of Cu for synthesis of multi-copper oxidase and/or formation of Fe oxides for catalysis, Mn deposits generally occur after BIF and GIF (Fig. 3). After Mn(II) oxidation, Mn(III, IV) oxides may undergo microbial reduction and silicification to precipitate Mn ore minerals [122].

Phosphorite deposits occur after Mn deposits [92], and their formation is related to enhanced continental weathering and input of dissolved P into stratified oceans. During massive BIF (GIF)/Mn deposition, P is mostly adsorbed onto the Fe and Mn mineral surface. After BIF/GIF and Mn deposition, there is sufficient free phosphorous to promote precipitation [92,125] via two scenarios: (i) formation of hydrogen sulfide from microbial sulfate reduction and its subsequent oxidation by sulfur-oxidizing bacteria to form polyphosphate intracellularly. Under an anoxic condition, sulfur-oxidizing bacteria hydrolyze polyphosphate to precipitate hydroxyapatite [126]; (ii) cyanobacterial bloom concentrates organic phosphorous and under anoxic conditions, sulfate-reducing bacteria decompose it to form inorganic phosphorous [127,128]. In either scenario, phosphorite formation requires a high level of sulfate, which explains an approximate correlation between seawater sulfate concentration [125] and phosphorite genesis [90] in a relatively oxic environment.

#### Stromatolite formation during the Boring Billion

After the GOE and formation of iron, manganese and phosphorous deposits, the Earth entered the so-called ‘Boring Billion’ period. This period is characterized by a constant O_2_ level [87], which is attributed to a low efficiency of photosynthesis due to a limited supply of nutrients into the oceans [129]. A noticeable event during this time period is the massive deposition of stromatolites [130]. Their formation mechanism involves several steps including production of adhesive compounds by diverse microorganisms that cement sand and other rocky particles to form microbial mats. Stromatolites occur in many different morph-types and are dominated by carbonate minerals. They first appeared in the Paleoarchaean (3.7 Ga) and reached peak abundance in the Mesoproterozoic, and gradually disappeared in the Phanerozoic [130]. The massive occurrence of stromatolites during this period is probably due to calcification of cyanobacteria, driven by oversaturation of carbonate minerals induced by CO_2_-concentrating mechanisms.

### The NOE and origination of metazoans

During the late Proterozoic, there was extensive soil formation from continental weathering, including a more-than-an-order-of-magnitude increase in the production of clay minerals [131] (Fig. 3b). The so-called ‘clay mineral factory’ leads to enhanced burial of organic carbon [131], a further rise in atmospheric O_2_ level (and therefore the NOE) and possibly the emergence of metazoans. However, the role of clay minerals in organic carbon burial and the NOE calls for further substantiation.

### Unique mineral–microbe interactions in the Anthropocene

The Anthropocene is a time period in which human activity has been the main driver for the changing environment. Greenhouse gas emission, disposal of heavy metals, and applications of antibiotics, plastics, concrete and synthetic fertilizers are some of the ways that human activity affects the environment. As a result, some microbial species become extinct, while new ones emerge. Likewise, human activity accelerates or slows mineral weathering. Therefore, mechanisms of mineral–microbe interactions are undoubtedly altered by human activity, and this represents a new area for future research.

## APPLICATIONS OF MINERAL–MICROBE INTERACTIONS

Studies of mineral–microbe interactions have many biotechnological applications. Here we review four research areas: (i) bioleaching and mineral fertilizers; (ii) remediation of heavy metals and organic pollutants; (iii) biosynthesis of novel materials; (iv) carbon sequestration.

### Bioleaching and mineral fertilizers

**Figure 4.**
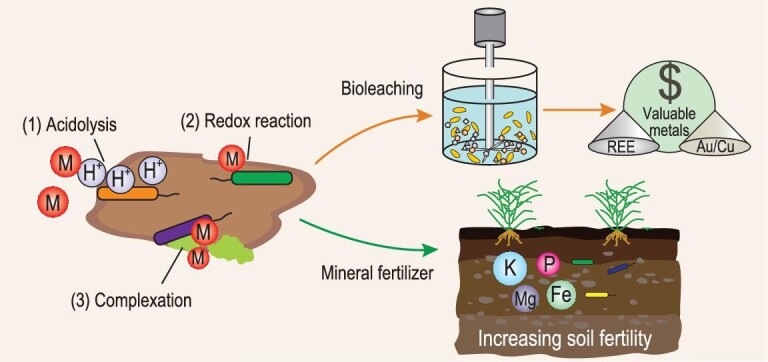
Application of mineral–microbe interaction in bioleaching and development of mineral fertilizers. The primary pathways in such applications include (1) acidolysis; (2) redox reaction; and (3) complexation. Bioleaching primarily refers to microbial extraction of valuable metals (e.g. Au, Cu and REE) from low-grade ores, mining wastes and tailings. Mineral fertilizer refers to microbial weathering of minerals and release of nutrient elements (e.g. K, Mg, P and Fe) to support crop growth.

Microbial weathering results in the mobilization of elements, which has important implications for resource recovery and development of mineral fertilizers. The primary pathways of elemental release from minerals include (Fig. 4): (1) acidolysis—microbial metabolic activities produce acidic substances including inorganic acids (e.g. sulfuric, nitric and carbonic acid), organic acids (e.g. oxalic and citric acids) and acidic EPSs, which lower the pH of their surroundings and promote the dissolution of minerals; (2) redox reactions—redox-sensitive elements in minerals/rocks can be either reduced or oxidized as part of microbial activity, and thus are released to the environment due to change of mineral solubility; (3) complexation—microbial metabolites such as siderophores and organic acids can form strong complexes with metals and therefore lead to their mobilization from solid minerals/rocks [12,132,133].

Bioleaching is an important process in mining (i.e. biomining) and metallurgy (i.e. biohydrometallurgy) that extracts valuable elements [e.g. Cu, Zn, Au and rare earth elements (REEs)] from low-grade ores, mining wastes and tailings (Fig. 4) [134]. The leaching efficiency is determined by the nature of ores/tailings (e.g. mineralogical composition and particle size), microbial species (e.g. metal tolerance) and physicochemical conditions (e.g. temperature, nutrients and pulp density). In addition, the operation method, i.e. either direct-contact or non-contact bioleaching, affects the bioleaching efficiency [132]. Microbes applied in the bioleaching process are selected according to the intrinsic properties of metal-bearing minerals [133]. Metals in sulfide ores/tailings are extracted using sulfur and iron oxidizers via acidic/oxidative dissolution, while those in oxidized lateritic ores (e.g. goethite) are released via organic-complexation/reductive dissolution [133].

Compared with chemical leaching, bioleaching is environmentally friendly and less costly, and has shown advantages in the recovery of metals from low-grade ores [132]. Certain microbes may even have the potential to selectively enrich certain REEs from the environment [83], possibly because of different distribution patterns of anionic functional groups on their surface. However, there are only a few successful examples at commercial scale, such as recovery of Cu and Co from sulfide ores [133]. One difficulty in the upscaling of the bioleaching process is the maintenance of active microbial activity under extreme conditions. To overcome this challenge, a mixed microbial consortium with complementary functions may result in a better performance than a pure culture. Biogeochemical models are likely powerful tools for guiding commercial-scale development, in which the heterogeneity of minerals, the variety of microbes, and microbial metabolites can be all taken into consideration.

In addition to bioleaching, microbial-induced dissolution of minerals plays a critical role in the development of sustainable agriculture, in which microbes can extract nutrients from minerals and rocks to support the growth of crops (Fig. 4) [135]. For instance, potassium-solubilizing bacteria and phosphorus-solubilizing bacteria could interact with nutrient-bearing minerals (e.g. feldspar and apatite), actively resulting in the release of K and P to increase soil fertility.

### Remediation of heavy metals and organic contaminants

Another major application of microbe–mineral interactions is remediation of heavy metals and organic contaminants. Heavy metal contaminants can be remediated from the environment via immobilization, while organic contaminants can be either oxidatively or reductively degraded.

**Figure 5.**
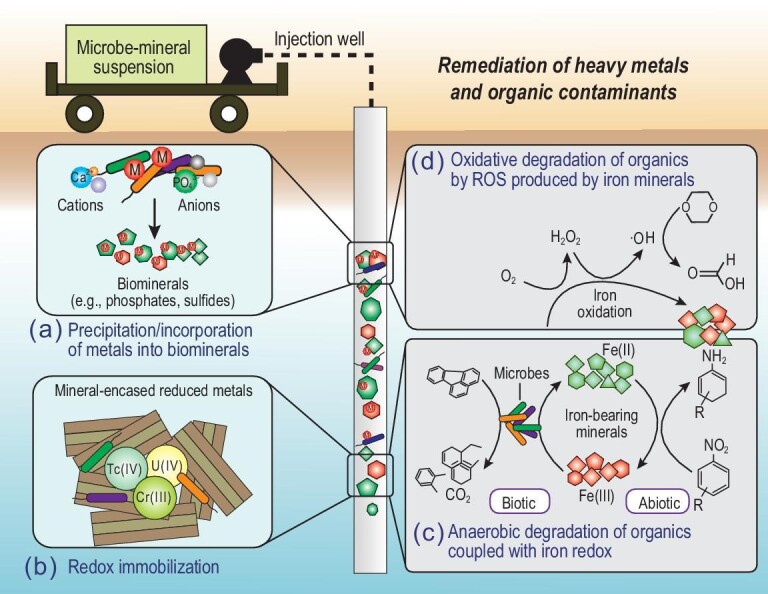
Environmental remediation of heavy metals and organic contaminants via microbe–mineral interactions. Toxic metals can be stabilized via injection of mineral–microbe suspensions to contaminant sites to result in either (a) precipitation/incorporation into biominerals or (b) formation of mineral-encased reduced metals through redox reactions. Organic pollutants can be degraded via (c) anaerobic oxidation coupled with Fe(III) reduction or anaerobic reduction coupled with Fe(II) oxidation, or (d) oxidative degradation by ROS (such as hydroxyl radicals **·**OH) produced from air oxidation of structural Fe(II) in minerals. M in (a) refers to metals. Reddish-brown symbols refer to oxidized Fe(III)-bearing minerals, and green symbols refer to reduced Fe(II)-bearing minerals. Organic compounds are schematic and do not specifically refer to certain compounds.

To remediate heavy metal contamination in the subsurface, microbe–mineral suspensions can be injected into contaminated sites to stimulate their interaction with heavy metals. The primary pathways of heavy metal immobilization include (Fig. 5): (i) precipitation/incorporation of metals into biominerals; and (ii) redox reactions. The principle of stabilization of heavy metals via biomineralization is the combination of acidic anions (e.g. S^2–^ and PO_4_^3–^) generated by microbial activity with toxic metal cations to precipitate poorly soluble minerals *insitu* (Fig. 5a). In some cases, cations (e.g. Ca^2+^) are amended to promote co-precipitation/incorporation of toxic metals into minerals [136]. The commonly used microbes in bioremediation of metals include urease-producing bacteria to form carbonates, phosphate-solubilizing bacteria to form apatite, sulfate-reducing bacteria to form sulfides and iron-oxidizing bacteria to form oxy(hydro)oxides. Biomineralization operates efficiently under a field condition, especially for radionuclides in the presence of certain oxidants such as nitrate that may otherwise inhibit bioreduction and/or induce re-oxidation of bioreduced metals [68]. For instance, uranium biomineralization via microbial phosphatase activity (i.e. degrading organic P into inorganic P resulting in the precipitation of metal-incorporated phosphate minerals) has been applied at the Oak Ridge site in the USA [68].

Most microbes are attached to the mineral surface and interact actively with minerals, resulting in the formation of reactive chemical species towards metal transformation [137]. The most common and reactive species is Fe(II) in the form of either surface-bound or structural Fe(II). Biogenic forms of Fe(II), typically formed from microbial Fe(III) reduction, reduce highly mobile and toxic metals such as U(VI), Cr(VI) and Tc(VII) to less mobile and toxic U(IV), Cr(III) and Tc(IV) [137] (Fig. 5b). The reduced metals that are protected by Fe(II)-bearing minerals, such as low-permeability clay minerals (Fig. 5b), should be more resistant to re-oxidation. However, the effectiveness of this approach in field-scale implementation depends on environmental conditions. One important consideration is the presence of ligands/organic matter, which affects the speciation of both reactants and products [137,138]. Clearly, more research is warranted to understand how the formation of metal-ligand complexes affects the thermodynamics and kinetics of the redox reaction. In particular, the environmental fate of organically complexed metals should be taken into consideration in the evaluation of the long-term stability of reduced heavy metal products.

Degradation of organic contaminants is often coupled with the Fe redox cycle. In an anaerobic environment, oxidative degradation of organic pollutants (e.g. aromatic contaminants) can be coupled with microbial reduction of Fe(III)-containing minerals [139] (Fig. 5c). This process has been observed in petroleum- or landfill-leachate-contaminated aquifers. Conversely, certain organic pollutants (such as nitroaromatic compounds and antimicrobial agents) can be reductively degraded by structural Fe(II) in minerals [137] (Fig. 5c). In oxic environments, organic pollutants, including trichloroethylene (TCE), 1,4-dioxane and enrofloxacin, can be efficiently degraded by ROS produced from the oxidation of structural Fe(II) [140] (Fig. 5d). In clay minerals, structural Fe can be redox-cycled multiple times to allow sustainable generation of ROS and degradation of such organic contaminants [141]. Therefore, the interaction between microbes and minerals holds great promise for remediation of heavy metals and degradation of organic pollutants.

### Biosynthesis of novel materials for various applications

Biogenic nanoparticles (such as those in iron, gold, silver, platinum, palladium, cobalt and selenium) have distinct physicochemical properties that have wide applications in materials, medicine, catalysis, biosensing and the environment. Nanomaterials of distinct sizes, shapes and bioactivity can be synthesized by a variety of microbes including bacteria, fungi, yeast and viruses. Relative to chemically synthesized materials under harsh conditions (temperature and pH etc.), these biosynthesized materials are more eco-friendly, and have lower toxicity and energy consumption [142].

Furthermore, biogenic carbonates have found applications in the construction industry. The principle is microbially induced carbonate precipitation (MICP) [143]. MICP is a particular application of BIM that uses urease and carbonic anhydrase (CA) to induce carbonate mineral precipitation. MICP has found wide application in the self-healing of cracks in concrete, and improvement of its mechanical durability and water absorption. A similar concept is used for manufacturing artificial coral reefs for recreation and restoration of marine life.

### Role of mineral–microbe interactions in carbon cycling

**Figure 6.**
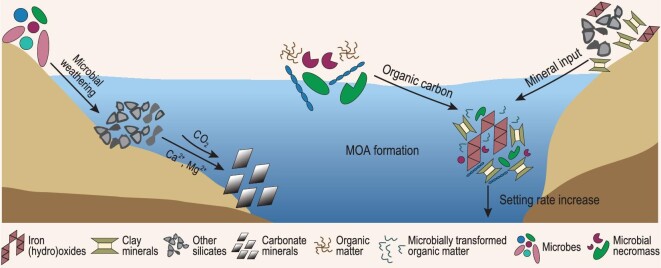
Schematic diagram showing the role of mineral–microbe interaction in the carbon cycle. Left side: microbial weathering of silicate minerals releases divalent cations to capture CO_2_ through carbonate formation. Right side: terrestrial input of fine-grained silts and clays forms aggregates with dissolved organic matter to form mineral-organic associations (MOAs), which increases the sedimentation rate of organic matter to marine sediment and results in carbon sequestration.

Climate change over a geological timescale is controlled by a balance between carbon release and sequestration. Mineral–microbe interactions play important roles in carbon sequestration via two mechanisms (Fig. 6): (i) microbially induced mineral weathering and carbonate formation, and (ii) organic carbon preservation by synergistic interaction of minerals and microbes. It is important to recognize that these two pathways may interact synergistically to enhance carbon sequestration. Various functional groups of organic matter may form certain chemical bonds with carbonate minerals, thus increasing the saturation state of carbonate minerals and the stability of both inorganic and organic forms of carbon [144].

#### Microbial weathering of minerals and carbonate formation

Weathering of silicate minerals, such as wollastonite, olivine, pyroxene, serpentine and brucite, releases Mg^2+^ and Ca^2+^ to capture CO_2_ through carbonate formation [145] (Fig. 6). Although the carbonation reactions are thermodynamically favorable, the kinetic rate is rather slow. Diverse microbes, including archaea, bacteria and fungi, accelerate carbonate formation through their role in mineral weathering.

Microbes accelerate silicate dissolution through production of excess proton, organic acids, siderophores and EPS [11]. Microbial consumption of major elements such as K and P lowers their aqueous concentrations, thus driving the dissolution of the host minerals due to the equilibrium effect. Fungi are considered to be effective in promoting mineral dissolution via their hypha [146]. Because fungi develop a strong adhesive force with target minerals through their hyphal tips, the close physical attachment allows significant pH reduction in the vicinity of cells, and biomechanical forces of hyphal growth breach the mineral lattice [147]. Consequently, an improved efficacy of cation release provides a promising approach for carbon sequestration in either engineered or natural processes.

Biological enzymes play a critical role in regulating mineral weathering and carbonate formation. One of the key enzymes is CA, which is ubiquitous in microorganisms and catalyzes the interconversion of CO_2_ to HCO_3_^–^ and H^+^, a rate-determining step for carbonate formation and dissolution. CA directly promotes carbonate formation by capturing atmospheric CO_2_ [148]. Microbes accelerate carbonate precipitation via a combination of extracellular activity of CA and ammonification to raise pH [149]. Moreover, other microbial processes, including photosynthesis, denitrification, sulfate reduction, and manganese and iron oxide reduction, also increase alkalinity and promote carbonate precipitation.

#### Organic carbon sequestration through synergistic interaction between minerals and microbes

Synergistic mineral–microbe interactions are crucial in sequestering large amounts of organic matter (OM). In soils and sediments, a large portion of organic carbon is associated with minerals, forming mineral-organic associations (MOAs) [150]. The long-term stability of such MOAs depends on the mechanisms of interaction between minerals and OM, including sorption, ligand and ion exchange, hydrophobic interactions, and aggregation. Because of its protection against microbial use, the formation of such MOAs decelerates OM decomposition and mineralization [151].

The role of minerals in sequestering OM is particularly profound in estuaries and river deltas that receive large amounts of terrestrial input of silts and clays (Fig. 6). In such dynamic environments, gradients of redox conditions and salinity promote clay flocculation, thus accelerating MOA sedimentation to ocean sediments (Fig. 6). Laboratory simulations have shown that the addition of clay minerals significantly increases the deposition rate of bacterial cells by several orders of magnitude [152], which should minimize cell lysis by viral activity, and is a likely reason for their preservation in marine sediments [153] and black shales [154].

However, microbial release and transformation of OM from MOAs also occur, which ultimately results in the release of a portion of OM to the atmosphere as CO_2_ [150]. The extent of OM release from MOAs depends on the mineral host, the strength of mineral-OM binding and environmental conditions [150,155]. As described above, microbes secret acids and metal chelators to acquire nutrients and energy from minerals. As a result, MOAs may be destabilized and OM is released/transformed. In redox-dynamic environments, MOA destabilization and OM release/transformation can be coupled with the redox cycling of Fe and Mn minerals [137,156–158].

Therefore, the fate of OM in MOAs depends on complex interactions among OM, minerals and microbes. During these processes, microbial activity consumes some original OM, and transforms it into new OM and microbial necromass. Microbial transformation enhances the chemical recalcitrance of OM through microbial carbon pump [159] and contributes to its preservation in oceans. However, in dynamic environments where OM, minerals and microbes interact with one another, there may be additional controls on the turnover of OM [160]. Microbial transformation of OM is the first step, which decreases size and increases polar and ionizable groups of OM, thus increasing water solubility [161]. The association of degradation products of OM with minerals through formation of MOAs is the second step, which favors the ultimate preservation of OM [162]. Therefore, minerals and microbes interact synergistically to preserve OM [163], forming a syntrophic mineral–microbe carbon pump. Future carbon cycle models are expected to incorporate both the mineral-specific (physical) and microbe-specific (chemical) protection mechanisms to more accurately predict the fate of OM in a climate change scenario.

## CHALLENGES AND FUTURE RESEARCH OPPORTUNITIES

Despite advances achieved through decades of research, major questions remain that may guide future research.

### Breaking traditional discipline boundaries: integrating mineral and microbial ecology

Although the roles of minerals in supporting microbial ecology are being increasingly recognized, their quantitative importance is yet to be established. This is often hindered by a lack of efforts to specifically consider the mineralogy effect when microbial ecological data (growth, diversity and functions) are correlated with environmental conditions through statistical analyses. Traditional culture media do not consider minerals as important sources of nutrients and energy, which may be one of the reasons for the low success rate of obtaining pure cultures (<1% culturable microbes). Mineral-based culture media should be more reflective of natural habitats and recover more microbial resources than the traditional culture media. Using the bait-fish analogy, it may be possible to ‘predict’ the colonizing community based on the physicochemical properties of the underlying minerals. Understanding the pathways of biomineralization is a continuing challenge. In particular, the ecological functions of biogenic minerals are speculative, especially for those minerals that require energy to form. An integrated effort that combines mineralogy and microbial ecologies should generate exciting new insights and drive the field forward.

### Searching for biological footprints in geological records: establishing mineral biosignatures

Distinguishing biogenic minerals from abiogenic ones is a continuing challenge. Mineral formation is a complex process, and there are multiple pathways to the formation of the same mineral. While it is difficult to determine the biogenic origin of a single mineral and to infer its functions, it is more informative to consider a syngenetic assemblage of minerals formed from a specific microbial function. Moreover, morphological, structural/textural and geochemical evidence should be sought to provide additional support of biogenicity and even to resolve specific physiological functions/metabolic pathways that are responsible for generating such biosignatures. Such mineral biosignatures may not only be used to explore early life on Earth but also to further the exploration of life on other planets.

### Building a bridge: linking laboratory mechanistic investigation to field observation

The discoveries of major mineral–microbe interaction events are often based on geological, geochemical and biomarker evidence, which may result in non-unique interpretation. Laboratory investigations usually focus on a mechanistic understanding under assumed conditions, which may be unrealistic. Thus, it is imperative to build a bridge between the two, such that: (i) the research question is discovered through field observations and guides laboratory simulations; (ii) experimental conditions are well-informed by field data; and (iii) laboratory results can be scaled up to explain field observations. Through an iterative approach of geological observations and laboratory simulations, past mineral–microbe interactions may be inferred.

One inherent complexity of this approach is the effect of time. Biogenic minerals are initially metastable and will undergo transformation over time. What is preserved in the geological record is the final mineralogical and microbial products. It is possible to manipulate conditions in the laboratory to mimic this transformation process. Another complexity is the evolving nature of geological conditions, and the fact that laboratory simulations are often carried out under a constant set of conditions. Therefore, it is important to vary the experimental conditions, along with numerical modeling, to account for the evolving nature of geological conditions. In this regard, it is important to make use of the mineralogy database from deep-time digital Earth (DDE) to look for how the composition and structure of a given mineral varies over time so that biogenic signals may be discerned.

### Learning from the past: manipulating mineral–microbe interactions for the benefit of humankind

The long history of mineral–microbe interaction has left many useful lessons that may be used to benefit society. The key to success is to manipulate mineral–microbe interactions and optimize the consequences of such interactions. For example, by manipulating mineral–microbe synergistic interactions, carbon could be sequestered and the global warming effect mitigated. Therefore, it is crucial to systematically understand carbon stability and transformation during mineral–microbe interactions, and their controlling mechanisms. Likewise, by making use of powerful genome-based metabolic modeling, it may be possible to dive into the vast reserves of mineral and microbe databases to develop predictive capabilities for maximizing resource recovery and environmental protection, as well as manufacturing novel materials.

## Supplementary Material

nwac128_Supplemental_file
