# Supplementary material for: A critical review of mineral–microbe interaction and co-evolution: mechanisms and applications
Source: Natl Sci Rev. 2022 Jul 4;9(10):nwac128. doi: 10.1093/nsr/nwac128 (PMC9522408; doi:10.1093/nsr/nwac128)
Supplement: nwac128_Supplemental_file [file nwac128_supplemental_file.docx]

**Supporting Information**

**A critical review of mineral-microbe interaction and coevolution:**

**mechanisms and applications**

Hailiang Dong^1,*^, Liuqin Huang^2^, Linduo Zhao^3^, Qiang Zeng^1^, Xiaolei Liu^1^, Yizhi Sheng^1^, Liang Shi^2^, Geng Wu^2^, Hongchen Jiang^2^, Fangru Li^1^, Li Zhang^4^, Dongyi Guo^1^, Gaoyuan Li^1^, Weiguo Hou^1^, and Hongyu Chen^1^

^1^ Center for Geomicrobiology and Biogeochemistry Research, State Key Laboratory of Biogeology and Environmental Geology, China University of Geosciences, Beijing 100083, China

^2^ State Key Laboratory of Biogeology and Environmental Geology, China University of Geosciences, Wuhan 430074, China

^3^ Illinois Sustainable Technology Center, Illinois State Water Survey, University of Illinois at Urbana-Champaign, Champaign, IL 61820

^4^ Department of Geology and Environmental Earth Science, Miami University, OH 45056, USA.

* Corresponding author: Hailiang Dong

China University of Geosciences, Beijing 100083,

Tel.: 86-010-82320969; Email: dongh@cugb.edu.cn

Revised for National Science Review

June 9, 2022

**Table S1. Minerals as sources of nutrients to shape microbial community**

| **Essential Elements** | **Mineral/Rock** | **Featured Microbes** | **Microbial Community Feature** | **Refs** |
| --- | --- | --- | --- | --- |
| P | Feldspars (with inclusions of apatite) | High microbial richness and diversity; Biofilm developed | | (1) |
|  | Apatite | At the phylum level, dominated by *Proteobacteria*, *Bacteroidetes*, *Acidobacteria*, and *Actinobacteria*; At the genus level, dominated by *Burkholderia*, *Pedobacter*, and *Chitinophaga*. | Abundant phylotypes mostly belong to genus with high mineral weathering efficacy | (2) |
|  | Apatite | Dominated by the class *Sphingobacteria* of *Bacteroidetes*  Harbored the archaea, *Thaumarchaeota* (Marine Group I) | High faith’s phylogenetic diversity | (3) |
| Fe | Biotite | Dominated by *Sphingobacteria*, γ-*proteobacteria*, and *Verrucomicrobia* | N/A |  |
|  | Ferrihydrite | *Bacteroidetes* tended to be enriched  significantly enriched the fungal *Sebacinales* and *Glomerales* | High microbial colonization | (4) |
| Fe, P | Anorthoclase (with inclusions of Fe-Ti oxide and fluorapatite) | High microbial biomass and the silicate matrix intensively etched | | (5) |
|  | Microcline (with fluorapatite) | Colonized to a less extent and lightly etched comparing to anorthoclase | |  |
|  | Anorthoclase (with Fe/P inclusions), white microcline (with P inclusion), apatite, and goethite | Under anoxic conditions, P- and Fe-rich minerals are heavily colonized by microbes | | (6) |
|  | Anorthoclase with Fe-oxides and apatite inclusions | Rod-shaped, cocci, filaments, helical polymers, and putative fungal filaments | Preferential microbial colonization, developed extensive biofilms | (7) |
|  | Apatite and obsidian | Enriched the genera *Mucilaginibacter*, *Burkholderia* and *Collimonas* | Abundant effective mineral-weathering bacteria | (8) |
| Fe, S | Bedrock in subglacial environment | Dominated by β- and γ-Proteobacteria | The microbial community structure resembles the structure of pyrite-, hematite-, and magnetite-inhabiting microbial communities | (9) |
| P, Na, Ca | Apatite and plagioclase | Dominated by *β-proteobacteria*, *α-proteobacteria*, *γ-Proteobacteria*, *Acidobacteria* | Small bacterial diversity relative to surrounding soils | (10) |
| Na, K | Obsidian | Dominated by *Burkholderia* | Contain effective P- and Fe- solubilizing bacterial isolates | (11) |
| Fe, K | Biotite | Abundant *γ-proteobacteria* when the medium lacks iron | High diversity when the medium lacks iron |  |
| Si, K, Ca, Mg | Granite | Dominated by classes *β-proteobacteria*, *Bacilli*, and *γ-proteobacteria*;  Genera *Pseudomonas*, *Serratia*, *Bacillus*, *Burkholderia, Chromobacterium,* and *Paenibacillus* were isolated | Abundant effective mineral-weathering bacteria | (12) |
| Mg, Fe, Ca | Labradorite; Olivine | Dominated by *Proteobacteria*, *Bacteroidetes*, *Acidobacteria*, *Actinobacteria*, *Verrucomicrobia* | High microbial richness and diversity | (13) |
| **Essential Elements** | **Mineral/Rock and Microbes** | **Physiological Response and Differential Gene Expression** | | **Ref** |
| Fe, Mg | Volcanic rock/ *Cupriavidus metallidurans* CH34 | Volcanic rocks resulted in increased rates of cell division and up-regulations of phosphate limitation proteins | | (14) |
| Fe | Basalt/ *Cupriavidus metallidurans* CH34 | Basalt resulted in the up-regulation of genes encoding putative components of ABC-type transporters, porins and extra-cytoplasmic solute receptors in the iron-limited conditions | | (15) |
|  | Biotite/ *Caballeronia mineralivorans* PML1(12) | The addition of biotite induced the differential genes and proteins expression to produce non-ribosomal peptide synthetase-independent siderophore | | (16) |
|  | Iron-rich vermiculite clay/ *Pseudomonas protegens* | High iron availability promoted the transcription of DAPG synthetic genes | | (17) |
| Fe, S | Pyrite/ *Methanococcus voltae* A3 | Pyrite induced the up-regulation of protein that were involved in Fe(II) transport | | (18) |
| K | Potassium minerals/ *Bacillus mucilaginosus* | The minerals induced the microbial production of high-protein extracellular polymers and proteins/exopolysaccharides that can enhance mineral weathering | | (19) |
| Cu | Borosilicate Glass/ *Methylosinus trichosporium* OB3b | Cu promoted the expression and activity of copper-containing pMMO, and enhanced CH_4_ oxidation rates of *M. trichosporium* OB3b | | (20) |
|  | Synthetic Cu-doped iron oxide and Cu-doped borosilicate glass/ *Methylosinus trichosporium* OB3b | Cu released from solid minerals stimulated pMMO synthesis and activity in *M. trichosporium* OB3b. Different solid-phase Cu geochemistry control the transcript levels and patterns of MMO. | | (21) |

| **Trace Elements** | **Mineral/Rock** | **Investigated Microbes** | **Related Enzymes and Functions** | **Ref** |
| --- | --- | --- | --- | --- |
| Mo | Silicate glass | *Azotobacter vinelandii* | Mo functioned as specific metal cofactors for nitrogenase enzyme. Cells secreted ligands (molybdophore) to uptake Mo from glass for N2-fixing | (22) |
| Ni | Banded iron formations | *Methanogens* | As the key metal cofactor in several enzymes of methanogens, Ni availability influenced the numbers of methanogen cells and methane production | (23) |
|  | Ni-containing silicate glass | *Methanothermobacter thermoautotrophicus*; *Methanobacterium formicium* | Methanogenic archaea leached Ni as necessary micronutrients from silicate minerals for growth | (24) |
| Cu | Malachite and Tenorite | *Methylosinus trichosporium* OB3b | Solid-phase Cu minerals readily support growth in *M. trichosporium* OB3b. The pMMO is sustained with CuCO_3_.Cu(OH)_2_ while sMMO activity is induced by low level of CuO | (25) |
| Fe | Ferrihydrite and hematite | *Anaeromyxobacter*  *Geobacter* | Robust nitrogen-fixing activity in ferrihydrite. Transcripts of *nifD* were induced by ferrihydrite and hematite | (26) |
|  | Pyrite | *Methanococcus voltae* strain A3;  *Methanosarcina barkeri* strain MS | Methanogens can catalyze the reductive dissolution of FeS_2_ and utilize dissolution products to synthesize simple and complex co-factors | (27) |

Notes: 1. pMMO: particulate methane monooxygenase; 2. sMMO: soluble methane monooxygenase.

**References**

1. Rogers JR and Bennett PC and Choi WJ. Feldspars as a source of nutrients for microorganisms. *Am Mineral* 1998; **83**: 1532-40.

2. Lepleux C, Turpault MP, Oger P *et al.* Correlation of the abundance of betaproteobacteria on mineral surfaces with mineral weathering in forest soils. *Appl Environ Microbiol* 2012; **78**: 7114-9.

3. Ahmed E, Hugerth LW, Logue JB *et al.* Mineral Type Structures Soil Microbial Communities. *Geomicrobiol J* 2016; **34**: 538-45.

4. Whitman T, Neurath R, Perera A *et al.* Microbial community assembly differs across minerals in a rhizosphere microcosm. *Environ Microbiol* 2018; **20**: 4444-60.

5. Rogers JR and Bennett PC. Mineral stimulation of subsurface microorganisms: release of limiting nutrients from silicates. *Chem Geol* 2004; **203**: 91-108.

6. Mauck BS and Roberts JA. Mineralogic Control on Abundance and Diversity of Surface-Adherent Microbial Communities. *Geomicrobiol J* 2007; **24**: 167-77.

7. Phillips-Lander CM, Fowle DA, Taunton A *et al.* Silicate Dissolution in Las Pailas Thermal Field: Implications for Microbial Weathering in Acidic Volcanic Hydrothermal Spring Systems. *Geomicrobiol J* 2013; **31**: 23-41.

8. Colin Y, Nicolitch O, Turpault MP *et al.* Mineral types and tree species determine the functional and taxonomic structures of forest soil bacterial communities. *Appl Environ Microbiol* 2017; **83**: e02684-16.

9. Mitchell AC, Lafrenière MJ, Skidmore ML *et al.* Influence of bedrock mineral composition on microbial diversity in a subglacial environment. *Geology* 2013; **41**: 855-8.

10. Uroz S, Turpault MP, Delaruelle C *et al.* Minerals Affect the Specific Diversity of Forest Soil Bacterial Communities. *Geomicrobiol J* 2012; **29**: 88-98.

11. Kelly LC, Colin Y, Turpault MP *et al.* Mineral Type and Solution Chemistry Affect the Structure and Composition of Actively Growing Bacterial Communities as Revealed by Bromodeoxyuridine Immunocapture and 16S rRNA Pyrosequencing. *Microb Ecol* 2016; **72**: 428-42.

12. Olsson-Francis K, Pearson VK, Schofield PF *et al.* A Study of the Microbial Community at the Interface between Granite Bedrock and Soil Using a Culture-Independent and Culture-Dependent Approach. *Advances in Microbiology* 2016; **6**: 233-45.

13. Wild B, Daval D, Beaulieu E *et al.* In-situ dissolution rates of silicate minerals and associated bacterial communities in the critical zone (Strengbach catchment, France). *Geochim Cosmochim Acta* 2019; **249**: 95-120.

14. Bryce CC, Le Bihan T, Martin SF *et al.* Rock geochemistry induces stress and starvation responses in the bacterial proteome. *Environ Microbiol* 2016; **18**: 1110-21.

15. Olsson-Francis K, Van Houdt R Fau - Mergeay M, Mergeay M Fau - Leys N *et al.* Microarray analysis of a microbe-mineral interaction. *Geobiology* 2010; **8**: 446-56.

16. Uroz S, Picard L, Turpault M-P *et al.* Dual transcriptomics and proteomics analyses of the early stage of interaction between Caballeronia mineralivorans PML1(12) and mineral. *Environ Microbiol* 2020; **22**: 3838-62.

17. Almario J, Prigent-Combaret C, Muller D *et al.* Effect of Clay Mineralogy on Iron Bioavailability and Rhizosphere Transcription of 2,4-Diacetylphloroglucinol Biosynthetic Genes in Biocontrol Pseudomonas protegens. *MOLECULAR PLANT-MICROBE INTERACTIONS* 2013; **26**: 566-74.

18. Payne D, Shepard EM, Spietz RL *et al.* Examining Pathways of Iron and Sulfur Acquisition, Trafficking, Deployment, and Storage in Mineral-Grown Methanogen Cells. *Journal of Bacteriology* 2021; **203**: e0014621.

19. Xiao B and Lian B and Shao W. Do Bacterial Secreted Proteins Play a Role in The Weathering of Potassium-Bearing Rock Powder? *Geomicrobiol J* 2012; **29**: 497-505.

20. Kulczycki E, Fowle DA, Kenward PA *et al.* Stimulation of Methanotroph Activity by Cu-Substituted Borosilicate Glass. *Geomicrobiol J* 2011; **28**: 1-10.

21. Knapp CW, Fowle DA, Kulczycki E *et al.* Methane monooxygenase gene expression mediated by methanobactin in the presence of mineral copper sources. *PNAS* 2007; **104**: 12040-5.

22. Liermann LJ, Guynn RL, Anbar A *et al.* Production of a molybdophore during metal-targeted dissolution of silicates by soil bacteria. *Chem Geol* 2005; **220**: 285-302.

23. Konhauser KO, Pecoits E, Lalonde SV *et al.* Oceanic Nickel depletion and a methanogen famine before the Great Oxidation Event. *Geochim Cosmochim Acta* 2009; **73**: A678-A.

24. Hausrath EM, Liermann LJ, House CH *et al.* The effect of methanogen growth on mineral substrates: will Ni markers of methanogen-based communities be detectable in the rock record? *Geobiology* 2007; **5**: 49-61.

25. Fru EC, Gray ND, McCann C *et al.* Effects of copper mineralogy and methanobactin on cell growth and sMMO activity in Methylosinus trichosporium OB3b. *Biogeosciences* 2011; **8**: 2887-94.

26. Masuda Y, Shiratori Y, Ohba H *et al.* Enhancement of the nitrogen-fixing activity of paddy soils owing to iron application. *Soil Sci Plant Nutr* 2021; **67**: 243-7.

27. Payne D and Spietz RL and Boyd ES. Reductive dissolution of pyrite by methanogenic archaea. *The ISME Journal* 2021; **15**: 3498–507.
